# Proposed Treatment Protocols for Facial Rejuvenation Using a Novel Absorbable Polydioxanone Monofilament Threadlift in Koreans: Empirical Perspectives of Aesthetic Physicians and Surgeons

**DOI:** 10.1093/asjof/ojaa049

**Published:** 2020-11-13

**Authors:** Doo-Yeoul Chang, Hyoung-Moon Kim, Tae Hwan Ahn, Sang Bong Lee, Hyoung-Jin Moon

## Abstract

**Background:**

Aesthetic physicians and surgeons should consider differences in anthropometric and anatomical characteristics between Asians and Caucasians in performing facial rejuvenation procedures using absorbable threadlifts in Koreans.

**Objectives:**

This paper was prepared to propose empirical treatment protocols for Korean aesthetic physicians and surgeons.

**Methods:**

A panel of 5 Korean experts on the aesthetic uses of an absorbable polydioxanone (PDO) monofilament threadlift (Mint Lift; HansBiomed Co. Ltd., Seoul, Korea), thus termed as “the Mint Consensus Group,” was convened to recommend practical guidelines for empirical treatment with the Mint Lift.

**Results:**

To summarize, our recommendations are as follows: First, the entry and exit points should be determined considering the anatomical characteristics of the face (level of evidence III). Second, treatment procedures may vary depending on indications (level of evidence III).

**Conclusions:**

Here, the authors propose empirical treatment protocols for facial rejuvenation using a novel absorbable PDO monofilament threadlift in Koreans. But more evidence-based efforts should be made to update the current treatment protocols.

**Level of Evidence: 4:**

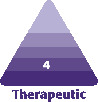

To date, surgical face-lifting has been frequently performed to excise the redundant skin for facial rejuvenation.^1,2^ But patients receiving surgical face-lifting are vulnerable to postoperative complications (eg, infection, skin necrosis, hematoma, seroma, nerve damages, and visible scars).^3,4^ It has been, therefore, imperative that minimally invasive facial rejuvenation techniques be developed; their merits include a lower morbidity and a rapid wound healing.^3^ Thus, novel nonsurgical facial rejuvenation procedures with volumizing effects have emerged. Their demerits, such as increased facial volume, unnatural contour, and visible shift of the gravity center to the lower one-third of the face, have remained problematic.^2,4-6^ Moreover, despite the efficacy of ablative or non-ablative resurfacing techniques in improving the skin surface, they have a lack of sufficient lifting of the underlying ptotic tissues.^7,8^

Patients with sagging and facial flaccidity are recommended to receive a threadlifting whose merits include decreases in time to recovery and incisions.^6,9^ Therefore, a threadlifting has been considered an alternative to conventional types of surgical face-lifting.^9,10^

In 2002, Sulamanidze et al^11^ first reported treatment outcomes of threadlifting using polypropylene barbed anti-ptosis sutures. Since then, diverse types of threadlifting procedures have been developed.^12^ Particularly in Asia, polydioxanone (PDO) threadlifts have recently become commercially available and then used for minimally invasive facial rejuvenation procedures.^13-16^

Classified as a class IV medical device, the Mint Lift (HansBiomed Co. Ltd., Seoul, Korea) is a violet-colored, absorbable PDO monofilament threadlift with a wire length of 43 cm and a US Pharmacopeia (USP) size of 0. Its properties include a transparency seen on postoperative month 1, bidirectional helical barbs providing an initial strong skin anchorage without an attachment of the yarn to the needle. Moreover, it is inserted initially using a curved needle (5/8) and then later using an 18-G blunt cannula, for which the disposable, external trocar is concomitantly used.^17-19^ Diverse types of the Mint Lift products, such as the Mint Lift 43, 17, Fine and Easy, are available to meet the needs of aesthetic physicians and surgeons *as well as* patients.^19^

Given the above background, we conducted this study to propose treatment protocols for facial rejuvenation using the Mint Lift products in Koreans.

## METHODS

### Study Setting

A panel of 5 Korean experts on the aesthetic uses of an absorbable PDO monofilament threadlift (Mint Lift), thus termed as the “Mint Consensus Group,” was organized on November 7, 2018. The “Mint Consensus Group” is composed of 5 aesthetic physicians and surgeons, whose specialty areas include facial and body contouring, urology, facial plastic surgery, otorhinolaryngology, and obstetrics and gynecology. All of them were men with a mean age of 48.20 ± 4.96 years old. The number of cases for which they had performed facial rejuvenation procedures by the end of 2018 ranged between 50 and 4000.

In April 9, 2019, the Mint Consensus Meeting was held to recommend empirical treatment protocols for facial rejuvenation using the Mint Lift.

To propose the current treatment protocols, we performed a review of the previous published studies about facial rejuvenation using a threadlift. Moreover, a formal discussion was convened to reach a consensus on the best clinical practices for facial rejuvenation using the Mint Lift between the members of the “Mint Consensus Group.” Then, their opinions were compiled by collecting personal experiences and opinions through discussions. The current study was conducted during November 7, 2018, and April 9, 2019.

### Ethical Statement

The current study was conducted in compliance with the relevant ethics guidelines; all procedures described herein were performed in accordance with the 1964 Declaration of Helsinki and its later amendments or comparable ethical standards. The current study was approved by the Internal Institutional Review Board of the Korea National Institute of Bioethics Policy (2020-03-637-172). But a written informed consent was waived due to the retrospective nature of the current study.

## RESULTS

### Anatomic Considerations in Determining the Entry and Exit Points

For facial rejuvenation procedures using the Mint Lift, the entry and exit points are determined considering the anatomic characteristics of the face, as previously described.^12,17-19^

### Indications for Treatment Procedures Using the Mint Lift Fine

**Figure 1.**
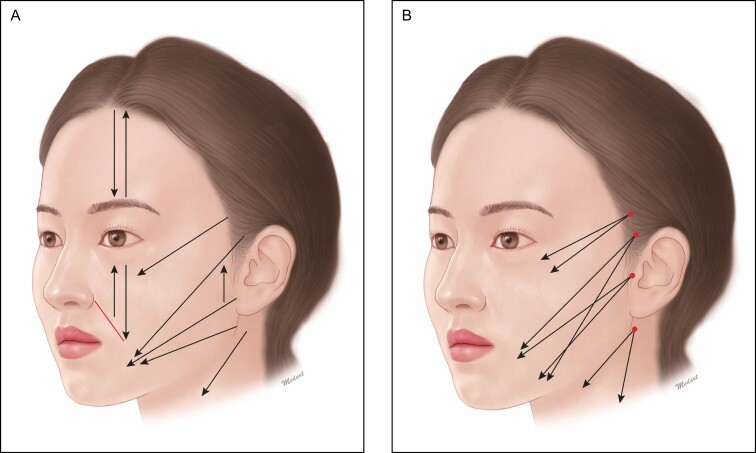
Use of a non-fixation type, bidirectional cog thread. (A) Also known as a floating threadlift, the Mint Lift Fine is advantageous in that there are no limitations in the entry site. (B) The entry point may be located in a limited scope because of the possible occurrence of dimpling.

The Mint Lift Fine can be inserted in any entry points determined on the face because of its user-friendly interface that is irrespective of vectors and a lack of possibility of its causing dimpling at the entry points (Figure 1A). But there is a possibility that the entry point may be weakened, for which it would be mandatory to tie a knot in the threadlift. In addition, the entry point may be located in a limited scope because of the possible occurrence of dimpling (Figure 1B).

#### Indications: Nasolabial folds, Nasolabial Fat and Midfacial Rejuvenation

In patients with a poorly developed zygomatic arch, the midface lifting is performed using the vector perpendicular to the nasolabial folds. That is, such vector is horizontal to that used for temporal anchoring. It, therefore, becomes possible to perform the procedure by attaching the threadlift to the periphery of the eye without lifting the eye corner. This is advantageous in preventing the formation of the lifted eye corner which most of the Koreans dislike and the rigid boundary formed by the zygomatic ligament.

**Figure 2.**
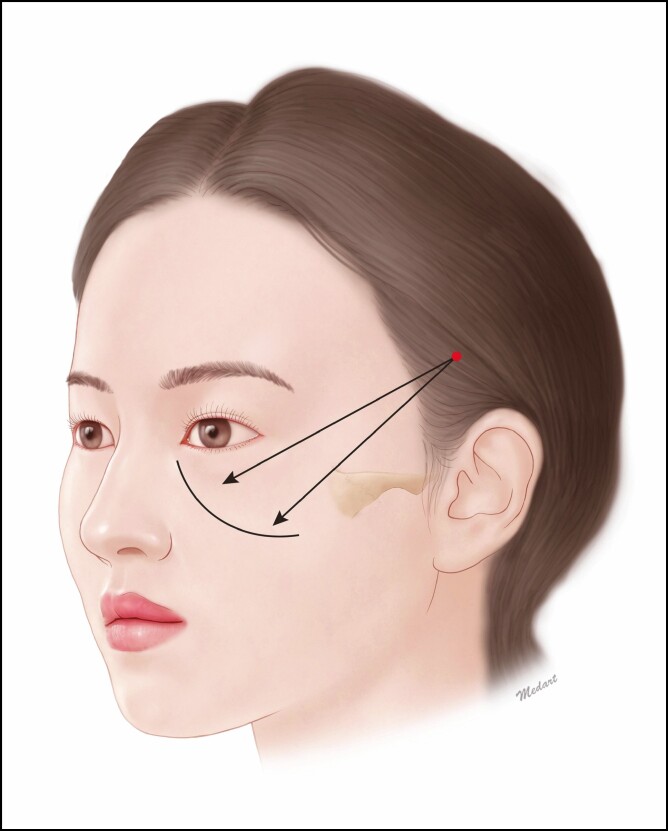
Design of midfacial lifting. The entry point is determined below the temple, and the exit one is located within the lateral canthus but not displaced to the zygomatic cutaneous ligament.

The entry point is determined below the temple, and the exit one is located within the lateral canthus but not displaced to the zygomatic cutaneous ligament. Due to an abundant presence of the fibrous tissue in the lower face, it is highly movable. This may cause dimpling when the cog is stuck (Figure 2).

The depth of the insertion of the threadlift deserves special attention because of a thin layer of the subcutaneous fat in patients for whom the exit point is located near the maxilla. A slight deeper insertion of the threadlift may cause it to enter the premaxillary space, which may produce poor treatment outcomes, such as a thickening of the premaxillary space.

**Figure 3.**
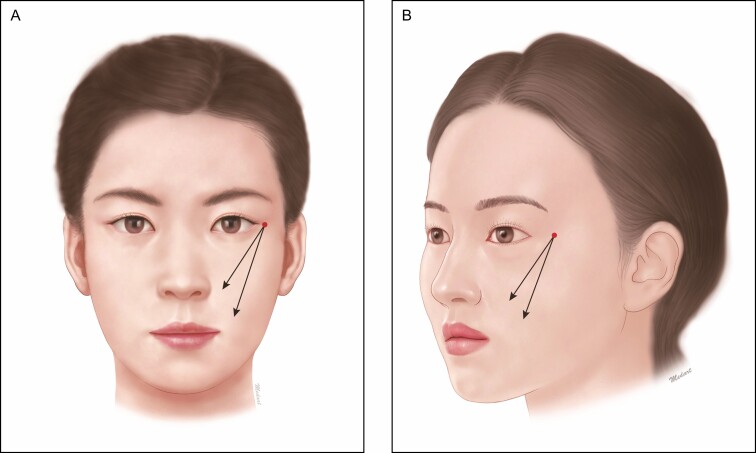
Modified design for patients with a well-developed zygomatic arch. (A) The entry point be determined around the inner zygomatic arch and the exit one be located above the nasolabial folds. (B) Use of short bidirectional cog thread or zigzag cog thread is recommendable for patients with a short target area.

In patients with a well-developed zygomatic arch, the entry point cannot be determined on the temple. This is because treatment effects cannot be achieved involving the inner superficial muscular aponeurotic system (SMAS) in such patients. It is, therefore, recommended that the entry point be determined around the inner zygomatic arch and the exit one be located above the nasolabial folds (Figure 3A). Moreover, the use of short bidirectional cog thread or zigzag cog thread is recommendable for patients with a short target area (Figure 3B).

#### Indications: Jowl Fat, Marionette’s Line, and Lower Facial Rejuvenation

In patients with jowl fat, Marionette’s line and those who are in need of lower facial rejuvenation, the Mint Lift Fine is inserted in the entry site around the zygomatic arch before the temporomandibular joint (TMJ) through a lateral ear approach. Then, the exit point is determined on the jowl fat and the knot is tied (Video 1). This is such a simple, efficient technique that can be performed by even inexperienced aesthetic physicians and surgeons, whose advantages include the following: First, a postoperative recovery period is relatively shorter. Second, even the midcheek hollowness can be effectively corrected by lifting the sagging fat to the midface with the use of a bidirectional cog threadlift. This may eventually contribute to forming a more natural egg-shaped face. Third, the entry site is less affected by the jaw movement because it is located proximal to the TMJ, which leads to a decrease in the degree of mechanical stimulation of barb to the tissue, arising from the jaw movement, followed by stabilization of the fixation of threadlift at the entry point. Fourth, because the entry point is determined above the zygomatic ligament, the threadlift can be fixed more stably through the application of the traction force from the zygomatic ligament. A knot is tied concomitantly with the procedure. This may sustain treatment effects by increasing the degree of tissue lifting. Fifth, from anatomical perspectives, the entry site is located remote from the pathway of the superficial temporal artery and then determined on the region where that of the temporal branch of the facial nerve is deeply present. Therefore, vital vessels and nerves are left intact. Sixth, patients can return to their daily lives even immediately after the procedure because of a relatively shorter recovery period.

**Figure 4.**
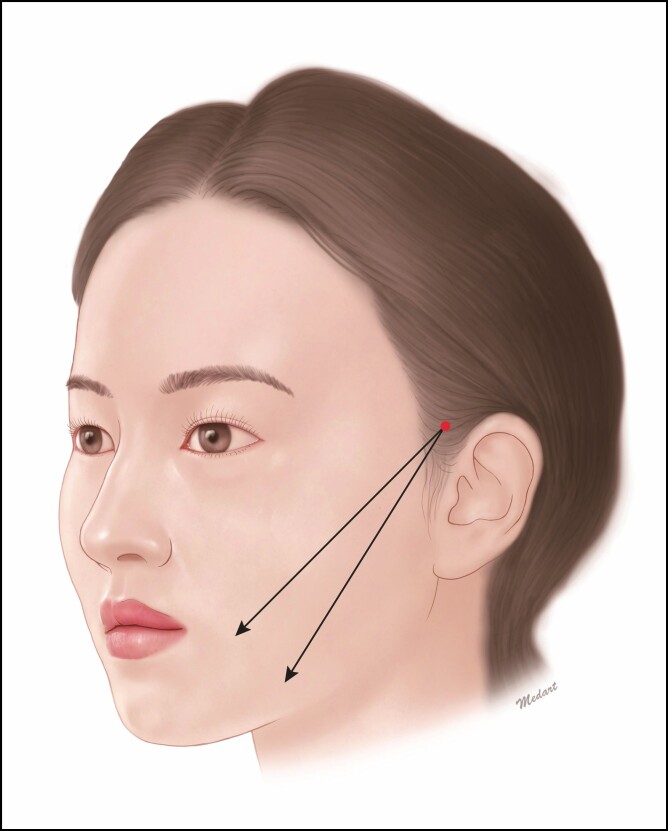
Location of the entry site below the ear or on the tragus. To determine the vector from the entry point to the exit one, the former is located on the zygomatic arch and the latter is determined in the jowl.

To determine the vector from the entry point to the exit one, the former is located on the zygomatic arch and the latter is determined in the jowl. If necessary, the entry site may be determined below the ear or on the tragus. Considering that the high SMAS face-lift technique is preferred for surgical lifting procedure, however, we assume that the entry site should be determined above the ear (Figure 4).

### Indications for Treatment Procedures Using the Mint Lift Easy

For the procedure using the Mint Lift Easy, the entry point is determined in the platysma-auricular ligament below the ear and the exit one is located in the jowl and lateral to the ear. Both the vertical and horizontal vectors are used to smoothly lift the tissue to the midline of the target site.

#### Indications: Rejuvenation of Jaw and Neck

For the rejuvenation of jaw, causes of age-related sagging should be checked. These include adipogenesis, sagging of the muscle, and development of salivary glands. In patients with sagging of the muscle, the double-jaw procedure has been the most effective treatment modality. A liposuction has been frequently performed for the rejuvenation of jaw, although the threadlifting is recommended as a more effective treatment modality in patients with severe sagging of the muscle. An accurate differential diagnosis should be made before the recommendation of the optimal treatment modalities.

A hammock procedure is performed using a fixation type bidirectional cog thread, for which the entry site is determined in the central part of the jaw and the exit point is bilaterally located in the outside of the jaw. It is differentiated from other procedures in that it is performed to fix both sides of the jaw and to pull its central part, without simply pulling the tissue, for the purposes of exhibiting hammock-like effects (Video 2).

**Figure 5.**
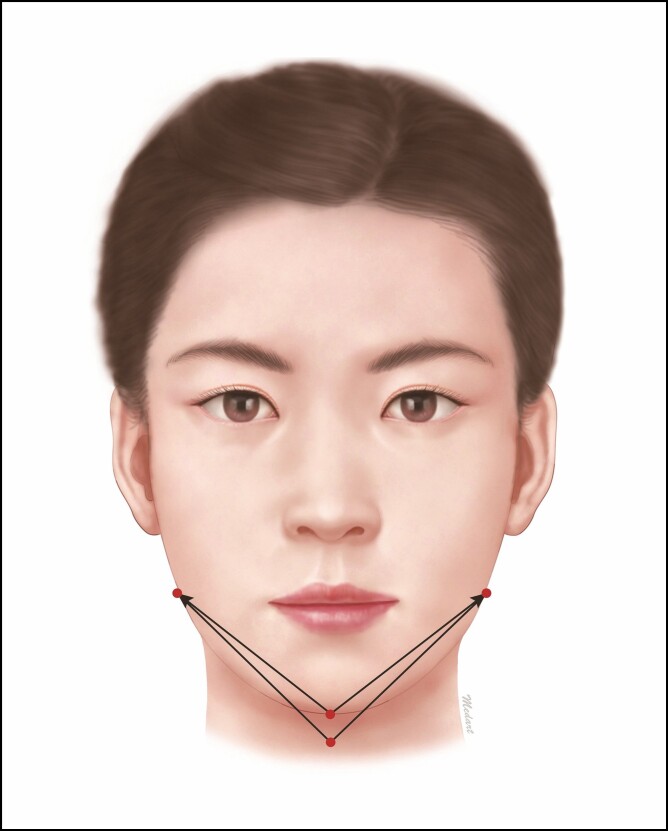
Hammock procedure. In a hammock procedure, the entry point located in the central part of the jaw has an effect in pulling the sagging tissue as it also serves as the exit one.

In conventional types of a fixation type, bidirectional cog thread is used to tract the tissue in the central part of the jaw, thus causing its bulging. But a hammock procedure is characterized by the traction of sagging tissue without causing its bulging. Therefore, the entry point located in the central part of the jaw has an effect in pulling the sagging tissue as it also serves the exit one (Figure 5).

As described above, a hammock procedure is an effective modality in patients with sagging of the muscle accompanied by a poorly developed fat tissue. Its treatment goal is to lift the platysma layer. In patients with slightly sagging of the fat, however, the superficial insertion of the threadlift in the entry point may effect in lifting the fat layer. Moreover, patients with an excessive amount of fat may also be treated with monofilament threadlift.

Patients are commonly placed in a cervical extension position, which is disadvantageous in that there is an increase in the degree of tension applied to the SMAS. This also leads to an increase in the degree of tension applied to the platysma muscle, which may lower the degree of effects in lifting it despite the traction of it. It is, therefore, recommended that patients be placed in such a position that the cervical spine is slightly flexed so that there may be a decrease in the degree of tension applied to the platysma muscle.

**Figure 6.**
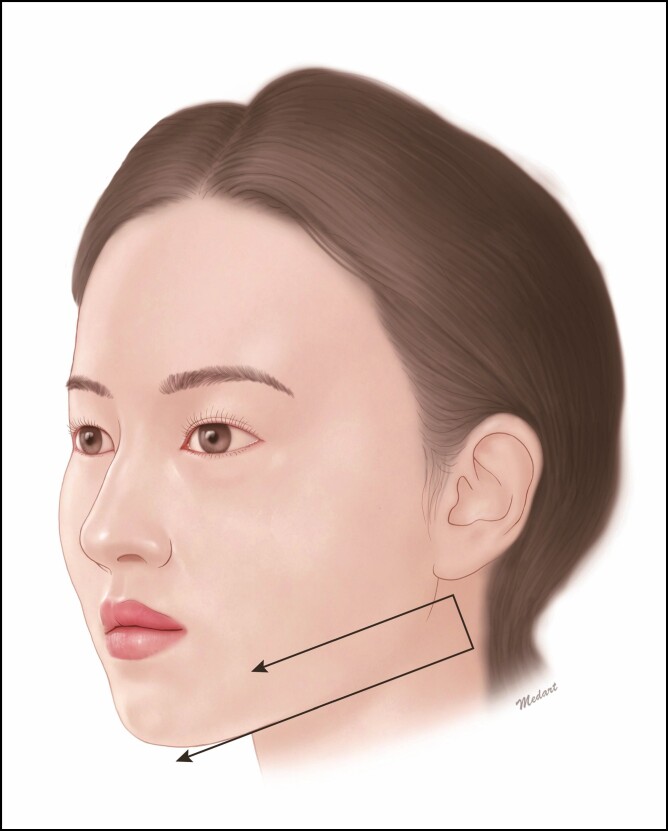
Staple-shaped procedure below the ear. The entry point is located in the platysma-auricular ligament below the ear and the exit one is determined in the jowl on one side and the supraplatysmal fat.

**Figure 7.**
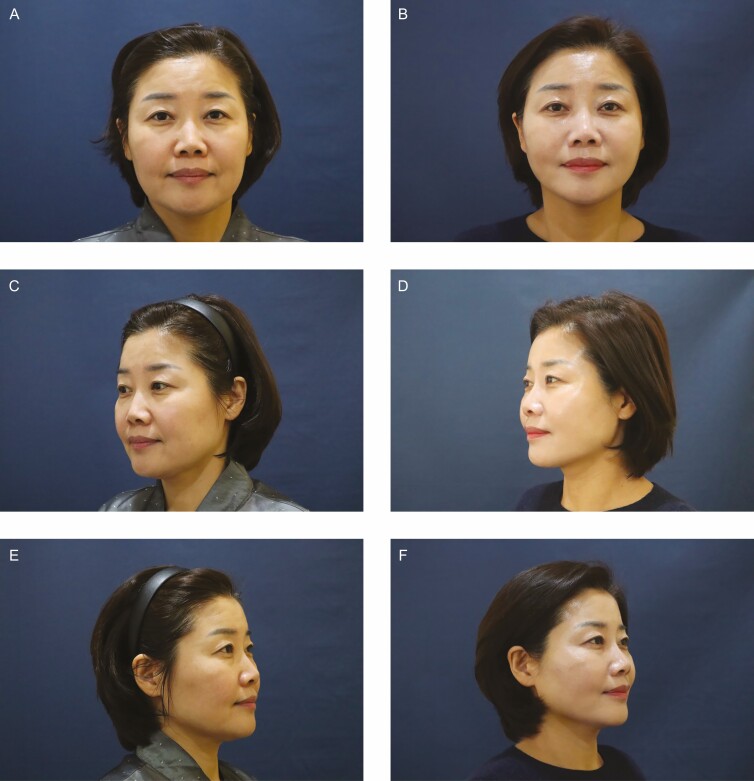

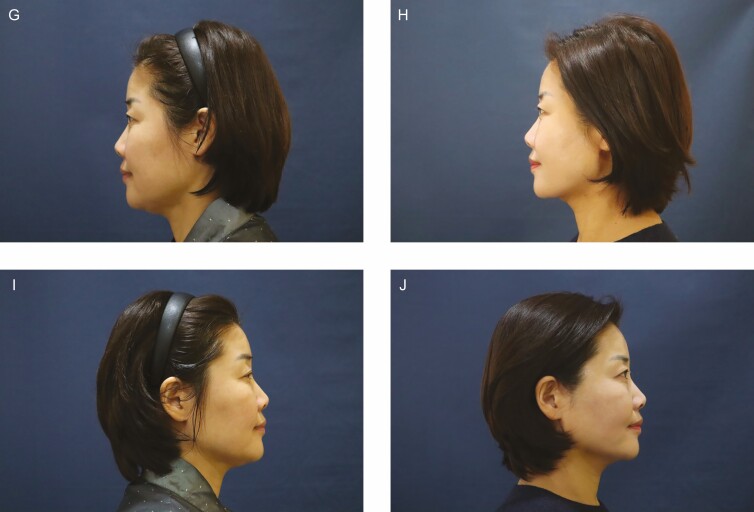
Illustrative case. A 51-year-old woman visited us with a chief complaint of sagging jowl, who received the treatment using the Mint Lift Fine for the midface and lower face. The patient also received the treatment using the Hammock procedure for the chin. At 6 months postoperatively, the patient achieved aesthetic improvements in nasolabial folds and Marionette lines by lifting ptosis of the superficial fat, such as nasolabial fat and jowl fat, but presented with no complications. Moreover, the patient also achieved a well-defined mandibular border by lifting the supraplatysmal fat. (A, B) On the frontal view, the patient postoperatively showed an oval-shaped face after achieving aesthetic improvements in Marionette lines by lifting the jowl fat. (C, D) On 45°-lateral view and (E, F) lateral view of the left side of the face and (G, H) 45°-lateral view of the right face, the patient achieved aesthetic improvements in the nasolabial crease by lifting the nasolabial fat. Moreover, the patient showed a well-defined mandibular border. (I, J) On the lateral view of the right side of the face, the patient showed a well-defined mandibular border by lifting the supraplatysmal fat.

A staple-shaped procedure is performed involving the jowl; it is a useful modality with the Mint Lift Easy in improving the mandibular contour. At the outpatient clinic, we commonly encounter patients without sagging of fat whose major concerns are improvement of the mandibular contour rather than that of the mandibular ptosis. These patients may be indicated in a staple-shaped procedure; the entry point is located in the platysma-auricular ligament below the ear, and the exit one is determined in the jowl on one side and the supraplatysmal fat (Figure 6). Illustrative case is presented in Figure 7.

## DISCUSSION

The advantages of minimally invasive facial rejuvenation techniques include shorter operation time, office-based treatment procedures, and fewer perioperative complications.^20,21^ Despite the availability of both absorbable and nonabsorbable threadlifts, the latter is disadvantageous in that it is permanently left in the tissue and then palpated on the skin. Patients receiving nonabsorbable threadlifts may be vulnerable to complications such as skin dimpling or suture extrusion. Therefore, the use of absorbable threadlifts has been preferred.^15,22,23^

With the emergence of PDO threads, great advances have been made in facial rejuvenation techniques using absorbable threadlifts.^16,21,23,24^ Of the absorbable PDO threadlifts, Mint Lift has been reported to be an effective, safe one.^12,17-19,24-26^ From empirical perspectives, treatment effects are sustained during a maximum period of approximately 8 months, for which further long-term follow-up studies are mandatory. Moreover, facial rejuvenation procedures using the Mint Lift require no overcorrection.

To summarize, our recommendations are as follows:

(1 ) The entry and exit points should be determined considering the anatomical characteristics of the face (level of evidence III).^12,17-19^
(2) Treatment procedures may vary depending on indications (level of evidence III).^12,17-19^ But this deserves further evidence-based studies.

## CONCLUSION

Here, we propose treatment protocols for facial rejuvenation using a novel absorbable PDO monofilament threadlift in Koreans. But more evidence-based efforts should be made to update the current treatment protocols.
